# Notes from the field: a case of hepatic failure and reflections on current status of healthcare of Syrian refugees in Lebanon

**DOI:** 10.1186/s13031-018-0149-x

**Published:** 2018-04-09

**Authors:** Salim M. Saiyed, John Kahler, Mutaz Kalabani

**Affiliations:** 1 Division of Health Sciences Informatics, Johns Hopkins School of Medicine, Baltimore, MD USA; 2 CaroMont Health, Gastonia, North Carolina, USA; 3 MedGlobal, Chicago, IL USA; 4 School of Medicine, Jordan University Of Science and Technology, Irbid, Jordan

**Keywords:** Non-communicable diseases (NCD), Syrian crisis, Refugee health, Health system in conflict, Displacement, Non-governmental organization (NGO), Lebanon

## Abstract

Recent trends and research suggest health conditions of the Syrian refugees in Lebanon are deteriorating. The following case study highlights some of the problems that refugees encounter seeking health care services in Lebanon. A coordinated response from Non-governmental organization (NGO) with intense focus on non-communicable disease (NCD) is needed to avert further worsening of health conditions on the ground.

## Main

A seven-year-old Syrian refugee girl presented with weakness, loss of appetite and intermittent fever. She had been well until nine months prior to presentation. After two weeks, her abdomen began to swell and she had change in her eye color. She was seen at outpatient clinic which primarily services Syrian refugees. Her initial physical revealed normal vital signs; she was clinically jaundiced and had ascites. She was transferred to a local hospital for evaluation. Initial Laboratory evaluations: SGOT 430 U/L; SGPT 155 U/L; Alk Phos 354; LDH 374; Total Bilirubin 5.53 mg/dl and Direct Bilirubin 2.30; Albumin 2.30 g/dl; CRP 14.7 mg/l; Hg 10.6 g/dl; Ceruloplasmine 0.054 g/l (normal 0.204–0.407) After 2 days in the hospital and 2 episodes of peritoneal dialysis, she was discharged for post-hospitalization follow up and seen by our mobile clinic. At the time, a presumptive diagnosis of Wilson’s Disease with acute hepatic failure was made. A recommendation was made to refer the child to a tertiary medical center for further evaluation and liver biopsy for confirmatory diagnosis. The family could not afford the further diagnostic work up so decided to travel back to Syria to explore options. Unfortunately three weeks after later patient passed away. With appropriate diagnosis and treatment, the survival rate for Wilson’s disease is favorable.

Over one million Syrian refugees live scattered across Lebanon in informal random clusters without basic amenities on the outskirts of small villages in inaccessible areas [1]. It is estimated that at least 30% of the refugees lack access to adequate potable water or sanitation facilities. Funding for the health care of refugees is supplied by the UNHCR and International community and delivered by public services NGOs as well as the Lebanese private sector in a fee for service system.

In Bekka Valley we witnessed a camp that had been burned to the ground. With over five hundred refugees- we found that in fact, this was a Potemkin Village. Although the shelters had been replaced, nothing was replaced on the inside, including water or sanitation.

We recently returned from one of the over dozen medical missions taken over the past three years to the region providing care to the Syrian refugees in Lebanon, Turkey, Jordan and Greece. For a month we provided medical care to the adult and pediatric patients in mobile clinics and ambulatory centers across Lebanon.

There has been an ongoing and continuing deterioration in the health status of refugees. Because of the lack of basic WASH needs, a majority of children have diarrhea and dehydration. Because of food insecurity, infants and children are failing to thrive. The refugee children’s dental status is appalling. Children with developmental disabilities, even when diagnosed early, have a paucity of resources for their ongoing care.

In adults we commonly saw acute decompensation of NCD such as heart failure that would otherwise require hospitalization due to lack of medical care. Accessing the private and fragmented Lebanese Health system remains a formidable challenge with a wide variety of coverage, if any [2]. We commonly witnessed cases like the one described above, causing increased morbidity and mortality and substantiated by other reports described by Coutts et al. [3].

The psychological conditions of adults and children are dire. Almost every child show signs of anxiety based illness: bedwetting, insomnia, headaches, and general “unhappiness”. Cultural, social barriers and general lack of awareness of psychological conditions pose additional challenges to treatment. Lack of psychological care coupled with the length of the crisis the situation and no immediate hope of future makes this crisis unprecedented [4].

NCDs frequently go untreated due to cost of receiving care [1, 5, 6]. Additional barriers including physical limitations, close access to health center, lack of public health care centers and transportation create barriers to access [5]. While many big and small medical NGOs with scores of medical professionals operate in the region, few standards exist on how to deliver and coordinate care [7].

The war continues and even though some refugees are beginning to be repatriated, the needs of the refugees in Lebanon and region in 2018 remain and will increase in the foreseeable future. As governments and relief agencies respond to the crisis, the focus should be to enhance delivery of primary care and improved education of local staff to increase their capacity [8]. Increasing health literacy of the refugees on NCDs and communicable diseases, early screening and mental health are needed to improve their resiliency. Novel approaches are desperately needed such as building infrastructure for universal electronic health record that can follow the refugees as they move across the region and receive care from variety of health care providers to keep continuity of care. The international medical community need to increase their response on the ground to alleviate the immediate and long term needs of the refugees.
